# Structural-Prior Deep Learning Network for Millimeter-Wave Radar Image Enhancement in Autonomous Driving Road Sensing

**DOI:** 10.3390/s26102976

**Published:** 2026-05-09

**Authors:** Hongyan Chen, Tonghui Huang, Yuexia Wang, Jiajia Shi, Zhihuo Xu

**Affiliations:** School of Transportation and Civil Engineering, Nantong University, Nantong 226019, China; 2433320006@stmail.ntu.edu.cn (H.C.); 2233320012@stmail.ntu.edu.cn (T.H.); shijj@ntu.edu.cn (J.S.)

**Keywords:** millimeter-wave radar, deep learning, structural prior, autonomous driving

## Abstract

**Highlights:**

**What are the main findings?**

- An adaptive Otsu-guided prior explicitly separates salient radar targets from background speckle.
- A continuous attention cascade and composite loss jointly optimize denoising and edge preservation.

**What are the implications of the main findings?**

- The architecture effectively mitigates over-smoothing degradation under severe multiplicative noise conditions.
- Superior structural preservation enables highly reliable zero-visibility perception for autonomous driving.

**Abstract:**

Millimeter-wave radar imaging plays an increasingly important role in autonomous driving road perception due to its robustness under adverse weather conditions. However, radar images are inherently contaminated by multiplicative speckle noise, which severely degrades structural continuity, weakens target boundaries, and limits the perception of road scenes and surrounding objects. To address this problem, this paper proposes a structural-prior deep learning network for millimeter-wave radar image enhancement. The proposed framework first introduces an adaptive Otsu-based masking strategy to extract salient scattering structures and generate a coarse image structural prior for subsequent restoration. Guided by this prior, the network performs progressive feature enhancement through a continuous attention mechanism that integrates residual channel attention, context-aware feature extraction, and convolutional block attention, thereby enabling effective multi-scale representation learning while suppressing signal-dependent speckle interference. In addition, a composite loss function is designed by combining logarithmic denoising gain, total variation regularization, and a β-index edge-preservation term to jointly improve noise suppression, spatial smoothness, and structural fidelity. The proposed method is evaluated on the synthetic UC Merced dataset under different noise intensities and via cross-domain inference on the real-world RADIATE millimeter-wave radar dataset for autonomous driving scenarios. Experimental results demonstrate that the proposed network consistently outperforms conventional filtering methods and representative deep learning baselines in terms of PSNR, SSIM, β-index, and ENL while providing a superior preservation of road structures, target contours, and scene geometry. Ablation studies further confirm the effectiveness of the structural-prior guidance and continuous attention design. Furthermore, the network achieves a rapid inference latency of 12.35 milliseconds. These results indicate that the proposed method provides an effective and robust solution for millimeter-wave radar image enhancement and offers practical value for downstream road-scene perception in autonomous driving environments.

## 1. Introduction

Millimeter-wave radar has become an important sensing modality for autonomous driving, surveillance, and environmental monitoring applications [1,2,3,4]. In particular, the rapid development of 4D mmWave imaging radar has triggered comprehensive reviews on autonomous driving perception pipelines, highlighting radar’s unique robustness and the growing importance of learning-based radar enhancement as a prerequisite for reliable downstream perception [5]. Compared with optical cameras and light detection and ranging sensors, millimeter-wave radar offers clear advantages in adverse conditions. Optical sensors are highly vulnerable to fog, rain, and poor illumination, while laser-based systems suffer performance degradation in heavy precipitation because of atmospheric attenuation. To mitigate these environmental degradations, significant research efforts have been dedicated to optical image restoration under adverse weather, such as exploring fuzzy priors in multi-mapping GANs for robust dehazing [6] and utilizing inverted haze density correction priors [7]. Although these advanced dehazing techniques effectively recover visual details for conventional cameras, optical systems still inherently struggle in extreme zero-visibility scenarios. By contrast, radar relies on longer-wavelength electromagnetic propagation and can, therefore, provide more reliable perception under all-weather and all-day conditions. This robustness makes millimeter-wave radar particularly attractive for safety-critical systems such as intelligent transportation. Moreover, enhancing radar quality directly benefits downstream detection in challenging driving scenarios, advancing 3D feature fusion [8,9], point-cloud integration [10], and LiDAR-radar BEV paradigms [11]. Beyond conventional CNNs, emerging physics-informed neural representations demonstrate that embedding physical priors is crucial for robust radar reconstruction under severe corruption [12,13].

Despite these advantages, millimeter-wave radar imaging faces a fundamental challenge: severe multiplicative speckle noise. Unlike optical image noise, radar speckle is signal dependent and originates from the coherent superposition of echoes returned by multiple independent sub-scatterers within the same resolution cell. Because the radar wavelength is often comparable to the macroscopic roughness of natural and human-made surfaces, the received backscatter contains random phase variations that produce constructive and destructive interference. As a result, speckle noise exhibits heavy-tailed and spatially varying characteristics, obscuring fine structures, weakening boundary contrast, and degrading the reliability of subsequent perception algorithms. In practical applications, such degradation may lead to missed detections or false alarms. Therefore, millimeter-wave radar denoising requires not only effective noise suppression but also the careful preservation of sparse high-reflectivity targets and structural boundaries. In this context, the introduction of explicit structural priors [14] and adaptive feature recalibration is particularly important for robust radar image restoration. Notably, the challenge of lacking truly clean reference images has motivated a surge in recent research in coherent imaging despeckling, including blind-spot self-supervised learning [15], semi-supervised learning with multitemporal references [16], multitemporal self-supervised deep filtering [17], and learning from near-real datasets constructed via adaptive multilook strategies [18]. These studies consistently suggest that structure preservation and domain-gap robustness are central bottlenecks for deploying deep despeckling models in real-world radar sensing.

Traditional denoising methods, including local-statistics-based Lee and Kuan filters [19] and Speckle Reducing Anisotropic Diffusion [20], have shown certain effectiveness in suppressing radar speckle. However, these approaches usually depend on empirical parameter tuning and handcrafted assumptions about local statistics. In complex scenes, they often oversmooth textures and weak structures, which limits their ability to preserve target details. With the rapid development of data-driven restoration methods, deep convolutional models such as Denoising Convolutional Neural Network, denoted as DnCNN [21] and Fast and Flexible Denoising Network, denoted as FFDNet [22], have achieved strong performance on natural image denoising tasks, and deep convolutional networks have gradually become an important paradigm for radar image analysis [4,23]. In the SAR despeckling community, a variety of recent deep architectures have been proposed to improve structural fidelity, including residual scaling networks for PolSAR [24], prior-driven structural-preserving networks [25], multiscale feature enhancement networks [26], and multiobjective training strategies using generic statistical samples [27]. To better address coherent imaging, recent studies have further explored specialized designs, such as residual twin networks with multi-resolution attention [28] and joint frameworks for denoising and super-resolution [29]. More recently, adaptive noise estimation frameworks enhance robustness under unknown noise levels [30], while hybrid Transformer–CNN models improve long-range dependency modeling [31]. Additionally, loss designs tailored to coherent imaging statistics, such as logarithmic discrete cosine transform constraints, effectively alleviate over-smoothing [32]. Furthermore, managing the noise-structure tradeoff is a shared challenge across coherent modalities. For instance, feature-guided CNNs for ultrasound despeckling [33] demonstrate that explicitly integrating structural fidelity into the loss function is far more crucial than merely maximizing the peak signal-to-noise ratio. Meanwhile, generative models show strong potential for speckle suppression, including conditional diffusion tailored to Gamma noise [34] and diffusion-Transformer hybrids [35].

Attention mechanisms have also been introduced to improve feature representation and adaptive recalibration in denoising tasks [36,37]. Although these mechanisms can enhance informative responses, directly applying attention over the entire radar image is often inefficient for millimeter-wave radar data. Background regions are frequently dominated by dense speckle and thermal noise, so unselective feature enhancement may allocate excessive modeling capacity to clutter, rather than to meaningful target structures. In addition, recent transformer-based methods have reported promising restoration performance [38,39,40,41,42,43], but their computational cost and limited explicit modeling of multiplicative speckle remain obstacles for practical real-time radar applications.

Motivated by these observations, this paper proposes a Structural-Prior Deep Learning Network for millimeter-wave radar image denoising. While explicit structural priors are effective, the existing approaches are primarily designed for conventional radar or optical sensors. To address the specific requirements of autonomous driving, our key idea is to introduce a structural prior stage before deep feature refinement that is explicitly tailored to millimeter-wave radar images. Specifically, an adaptive Otsu masking mechanism is employed to separate salient targets from background clutter according to the intensity distribution of millimeter-wave signals. Based on this prior, a continuous attention flow is constructed by cascading a Residual Channel Attention Block, a Context-Aware Block, and a Convolutional Block Attention Module, enabling progressive multi-scale feature enhancement and noise suppression. To further balance denoising strength and structural fidelity, a composite loss function is designed by combining the logarithmic denoising gain loss, total variation regularization, and a β-index edge-preservation term.

The main contributions of this work are summarized as follows:

1. A novel architecture, termed the Structural-Prior Deep Learning Network, is proposed for millimeter-wave radar image enhancement. While general structural priors have been explored, our specific contribution is the integration of an adaptive Otsu masking strategy as an early hard-attention filter explicitly designed for the scattering characteristics of millimeter-wave radar images. By introducing this specific image structural-prior layer, together with continuous attention-based feature refinement, the proposed framework effectively enhances informative multi-scale representations while suppressing speckle interference.
2. A composite loss function is developed by jointly integrating the logarithmic denoising gain, total variation regularization, and a *β*-index edge-preservation term, so that noise suppression, spatial smoothness, and structural preservation can be optimized in a unified manner.
3. Extensive experiments on the synthetic UC Merced dataset and the real-world RADIATE dataset demonstrate that the proposed method consistently outperforms representative baseline approaches in terms of PSNR, SSIM, and *β*-index while achieving superior visual quality, structural fidelity, and road-scene interpretability.

## 2. Proposed Method

Unlike optical sensors, which operate with incoherent illumination, millimetre-wave radar (MWR) is an active coherent imaging system. Within a single resolution cell, the transmitted electromagnetic waves interact with numerous elementary scatterers. Since the surface roughness of typical targets is often greater than the radar wavelength, the backscattered echoes exhibit random phase variations. The coherent summation of these echoes at the receiver gives rise to both constructive and destructive interference, thereby producing the granular phenomenon commonly referred to as speckle noise [44,45,46].

Due to this coherent scattering mechanism, speckle cannot be modeled as simple additive white Gaussian noise. Instead, it is highly signal-dependent and governed by a multiplicative degradation model. Let $I_{\mathrm{noisy}},I_{\mathrm{clean}}\in R^{H\times W\times C}$ denote the spatial tensors representing the noisy and clean images, respectively. The discrete imaging process at spatial coordinate (x,y) is formulated as:

(1) $$I_{\mathrm{noisy}}\left(x,y\right)=I_{\mathrm{clean}}\left(x,y\right)\cdot \eta \left(x,y\right),$$

where η(x,y) represents the signal-dependent speckle noise component. For fully developed speckle, η is statistically modeled to follow a Gamma distribution with a shape parameter, *L* (Equivalent Number of Looks), and the unit mean E[η]=1. The probability density function (PDF) is given by:

(2) $$p\left(\eta \right)=\frac{L^{L}}{\Gamma (L)}\eta^{L-1}\exp\left(-L\eta \right),\;\eta \geq 0.$$

From a Bayesian perspective, restoring $I_{\mathrm{clean}}$ can be formulated as a Maximum A Posteriori (MAP) estimation problem:

(3) $${\hat{I}}_{\mathrm{clean}}=\arg\max_{I_{\mathrm{clean}}}\left[\log p\left(I_{\mathrm{noisy}}|I_{\mathrm{clean}}\right)+\log p\left(I_{\mathrm{clean}}\right)\right],$$

where $p(I_{\mathrm{noisy}}|I_{\mathrm{clean}})$ is the likelihood term derived from the Gamma distribution, and $p(I_{\mathrm{clean}})$ represents the complex structural prior of the radar targets. The proposed deep learning framework aims to implicitly learn this highly non-linear prior mapping $F_{\theta }:R^{H\times W\times C}\to R^{H\times W\times C}$ parameterized by network weights θ:

(4) $$\theta^{*}=\arg\min_{\theta }E_{I∼p_{\mathrm{data}}}\left[L_{\mathrm{total}}\left(F_{\theta }\left(I_{\mathrm{noisy}}\right),I_{\mathrm{clean}}\right)\right].$$

### 2.1. Proposed Deep Network Model

**Figure 1:**
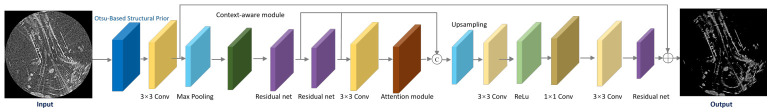
Overall architecture of the proposed network. An image structural-prior layer is introduced at the front end to provide prior structural guidance for the subsequent encoder–decoder framework. Intermediate features are fused through a skip connection with concatenation (denoted as “C”), while a long residual branch links the shallow features to the output through element-wise summation.

As shown in Figure 1, the proposed network adopts an encoder–decoder architecture under the guidance of an image structural-prior layer. Starting from the input radar image, the structural prior is first introduced to emphasize geometrical organization and salient structural patterns in the scene, thereby providing auxiliary guidance for subsequent feature extraction and reconstruction. Based on this prior-guided representation, the network progressively transforms the input features along the main encoding path and then reconstructs the denoised image through the corresponding decoding path.

A prominent characteristic of the proposed architecture is that the shallow features are propagated through two complementary routes. The first route corresponds to the main forward path, where features are successively processed by a series of intermediate blocks to accomplish hierarchical representation learning and image restoration. The second route forms a long residual branch that bypasses the deep transformation process and is directly connected to the network output through element-wise summation. Such a global residual design helps preserve low-level structural information, alleviates the loss of fine image details during deep feature transformation, and improves the stability of the final reconstruction.

In addition to the long residual branch, the network incorporates an intermediate skip connection between the encoder and decoder. As indicated by the symbol “C” in Figure 1, features from an earlier stage are fused with decoder features through concatenation before further refinement. This cross-stage fusion mechanism enables the decoder to reuse informative representations extracted in the encoder, enhances feature propagation across different depths, and facilitates the recovery of fine-scale structures that may otherwise be weakened in the progressive encoding process.

From the overall information flow shown in Figure 1, the network can be interpreted as a coordinated integration of three functional components. The first is the image structural-prior layer, which provides prior structural guidance for the subsequent restoration process. The second is the deep encoder–decoder backbone, which performs nonlinear feature transformation and image reconstruction through successive feature abstraction and recovery. The third is the combination of local and global skip pathways, which supports feature reuse, strengthens structural preservation, and improves the transmission of informative representations throughout the network.

By jointly exploiting prior structural guidance, deep feature transformation, cross-stage concatenation, and global residual learning, the proposed architecture is able to suppress undesired interference while maintaining salient target structures and boundary information in millimeter-wave radar imagery. This design is particularly beneficial for radar image restoration tasks, where the preservation of structural continuity and geometrical integrity is of central importance.

#### 2.1.1. Adaptive Otsu-Based Structural Prior Layer

In real-aperture millimeter-wave radar imaging, the acquired signals are heavily corrupted by multiplicative speckle noise, additive thermal noise, and ambient interference. To alleviate this severe background clutter before deep feature extraction, an adaptive Otsu-based masking stage is incorporated. Although classical Otsu thresholding assumes a bimodal Gaussian distribution, it can still provide a highly reliable structural prior despite the complex radar noise characteristics. The low-reflectivity background is dominated by thermal noise, which satisfies the baseline assumption, while high radar cross-section targets generate a significant intensity contrast relative to the surrounding clutter. This ensures that the inter-class variance strongly exceeds the speckle-induced intra-class variance, allowing strong structural scatterers to be reliably separated from background noise [47,48].

Let $T^{*}$ denote the optimal threshold maximizing the inter-class variance $\sigma_{B}^{2}\left(T\right)$ between high-reflectivity targets and the noise-dominant background:

(5) $$T^{*}=\arg\max_{T\in [0,255]}\left\{\omega_{0}\left(T\right)\omega_{1}\left(T\right){\left[\mu_{0}\left(T\right)-\mu_{1}\left(T\right)\right]}^{2}\right\},$$

where $\omega_{k}\left(T\right)$ and $\mu_{k}\left(T\right)$ denote the probability of occurrence and mean intensity of the *k*-th class, respectively.

Rather than producing an exact semantic segmentation, this procedure yields a coarse but robust structural prior. The structurally masked input tensor $I_{M}$ is thus defined as:

(6) $$I_{M}\left(x,y\right)=I_{\mathrm{noisy}}\left(x,y\right)\cdot I\left(I_{\mathrm{noisy}}\left(x,y\right)\geq T^{*}\right),$$

where I(·) is the indicator function selecting pixels with intensities exceeding $T^{*}$. Operationally, this indicator function acts as a binary spatial attention mask applied directly in the input domain. By performing an element-wise multiplication with the original noisy image $I_{\mathrm{noisy}}$, it explicitly isolates salient target features from dense background clutter. The resulting masked tensor $I_{M}$ then serves as the direct input $I_{in}$ to the initial convolutional layer of the subsequent feature extraction pipeline.

#### 2.1.2. Residual Channel Attention Layers

In deep convolutional networks, different feature channels hierarchically encode distinct types of information. For millimeter-wave radar images affected by multiplicative speckle noise, certain high-frequency channels are dominated by noise, while critical structural features are preserved in others. To address this, the Residual Channel Attention Block (RCAB) [49] is employed to adaptively recalibrate channel-wise responses, selectively emphasizing informative features and suppressing noise-dominated activations.

Let $F_{RCAB}\in R^{C\times H\times W}$ denote the input feature map. The RCAB first applies two successive 3×3 convolutions with ReLU activations:

(7) $$\begin{array}{cc} Z_{0} & =\delta (W_{3\times 3}^{\left(1\right)}*F_{RCAB}+b^{\left(1\right)}), \end{array}$$

(8) $$\begin{array}{cc} Z_{1} & =\delta (W_{3\times 3}^{\left(2\right)}*Z_{0}+b^{\left(2\right)}), \end{array}$$

where ∗ denotes convolution, and δ(·) is the ReLU function. To model inter-channel dependencies, a Channel Attention Block (CAB) is applied. The spatial information of each channel is aggregated via global average pooling:

(9) $$z_{c}=\frac{1}{HW}\sum_{i=1}^{H}\sum_{j=1}^{W}Z_{1}\left(c,i,j\right),\;c=1,\ldots ,C$$

A two-layer Multi-Layer Perceptron (MLP) with a reduction ratio, *r*, and a sigmoid activation produces the attention vector:

(10) $$s=\sigma \left(W_{U}\;\delta \left(W_{D}z+b_{D}\right)+b_{U}\right),\;s\in R^{C\times 1\times 1}$$

The recalibrated feature is obtained by element-wise multiplication with the attention vector, and a local residual connection is added:

(11) $$F_{RCAB}^{out}=F_{RCAB}+s⊗Z_{1}$$

This design enables the RCAB to selectively amplify meaningful channels while mitigating the influence of noise.

#### 2.1.3. Context-Aware Layers

The accurate extraction of multi-scale contextual features is essential for radar image restoration. Standard downsampling enlarges the receptive field but often results in loss of critical high-frequency details. To address this, we propose a Context-Aware Block (CB) that captures multi-scale context without reducing spatial resolution.

The CB processes the input feature map $F_{CB}$ through three parallel dilated RCABs with dilation rates d∈{1,2,4}:

(12) $$\begin{array}{cc} Z_{1} & =RCAB_{d=1}\left(F_{CB}\right), \end{array}$$

(13) $$\begin{array}{cc} Z_{2} & =RCAB_{d=2}\left(F_{CB}\right), \end{array}$$

(14) $$\begin{array}{cc} Z_{4} & =RCAB_{d=4}\left(F_{CB}\right) \end{array}$$

These multi-scale features are concatenated along the channel dimension:

(15) $$Z_{CB}=\mathrm{Concat}\left[Z_{1},Z_{2},Z_{4}\right]$$

A 1×1 pointwise convolution projects the concatenated features back to the original channel dimension, followed by a residual addition:

(16) $$F_{CB}^{out}=W_{1\times 1}*Z_{CB}+b+F_{CB}$$

This architecture effectively captures multi-scale contextual dependencies while preserving spatial resolution.

#### 2.1.4. Convolutional Block Attention Module

To further emphasize salient structures and suppress spatially varying speckle noise, we integrate a Convolutional Block Attention Module (CBAM) [50], which sequentially infers attention along both channel and spatial dimensions.

Given an input feature map, $F_{CBAM}$, the Channel Attention Module (CAM) first aggregates spatial information using both average and max pooling:

(17) $$\begin{array}{cc} F_{\mathrm{avg}}^{c} & =\frac{1}{HW}\sum_{i=1}^{H}\sum_{j=1}^{W}F_{CBAM}\left(c,i,j\right), \end{array}$$

(18) $$\begin{array}{cc} F_{\max}^{c} & =\max_{i,j}F_{CBAM}\left(c,i,j\right) \end{array}$$

These vectors are passed through a shared MLP and combined via a sigmoid activation:

(19) $$F^{\prime }=F_{CBAM}⊗\sigma \left(\mathrm{MLP}\left(F_{\mathrm{avg}}\right)+\mathrm{MLP}\left(F_{\max}\right)\right)$$

The Spatial Attention Module (SAM) then generates a spatial attention map by pooling along the channel axis:

(20) $$\begin{array}{cc} F_{\mathrm{avg}}^{\prime } & =\frac{1}{C}\sum_{c=1}^{C}F^{\prime }\left(c,:,:\right), \end{array}$$

(21) $$\begin{array}{cc} F_{\max}^{\prime } & =\max_{c}F^{\prime }\left(c,:,:\right) \end{array}$$

The concatenated 2D map is convolved with a 7×7 kernel and passed through a sigmoid function:

(22) $$F_{CBAM}^{out}=F^{\prime }⊗\sigma \left(f^{7\times 7}\left([F_{\mathrm{avg}}^{\prime }∥F_{\max}^{\prime }]\right)\right)$$

Here, ∥ denotes channel-wise concatenation, $f^{7\times 7}$ is a 7×7 convolution, and ⊗ indicates element-wise multiplication. This sequential attention mechanism allows the network to adaptively highlight task-relevant channels and spatial regions while suppressing background clutter.

#### 2.1.5. Overall Algorithmic Flow of the Attention Cascade

To synthesize the individual components described above, Algorithm 1 outlines the overall forward pass of the proposed network. Once the structural prior mask $M_{otsu}$ is applied to the initial feature representation, the resulting tensor undergoes a progressive refinement process. The cascade sequentially employs the RCAB for channel recalibration, the Context-Aware Block for multi-scale context aggregation, and the CBAM for joint spatial-channel enhancement. By structuring the network in this progressive manner, the initial structural guidance is naturally propagated through the deeper layers, allowing the model to effectively suppress speckle noise without compromising the geometrical integrity of salient targets.
**Algorithm 1:** Structural-prior-guided continuous attention network.
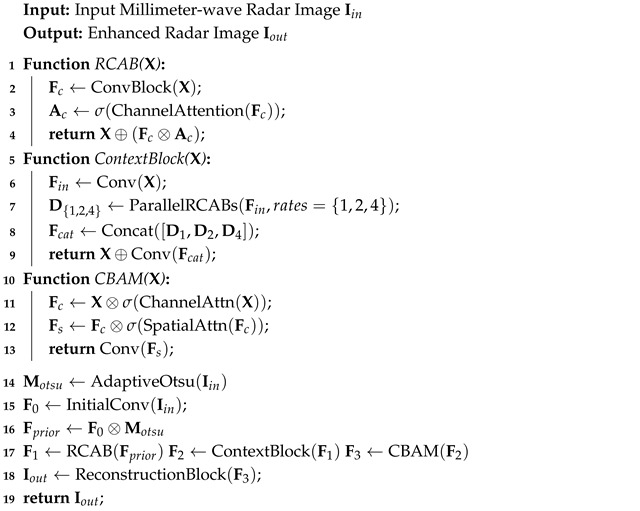


### 2.2. Composite Loss Function

To jointly optimize the noise suppression capability, spatial smoothness, and edge preservation, the objective function is formulated as a weighted combination of three complementary terms: a logarithmic denoising-gain loss, an isotropic total variation regularization term, and a β-index-based edge-preservation term.

Let $I_{\mathrm{noisy}}\left(x,y\right)$, $I_{\mathrm{clean}}\left(x,y\right)$, and $\hat{I}\left(x,y\right)$ denote the noisy input image, the clean reference image, and the reconstructed image predicted by the network, respectively, where (x,y) is the spatial coordinate, with x=1,2,…,W and y=1,2,…,H. For notational simplicity, we define the entire spatial domain of the image as Ω.

#### 2.2.1. Logarithmic Denoising Gain Loss

The Conventional Mean Squared Error (MSE) direct penalty may lead to suboptimal gradient behavior under signal-dependent multiplicative speckle. To better characterize the relative noise suppression ability and improve numerical stability, we measure the denoising process in terms of the residual-energy reduction ratio in a logarithmic domain.

Let ${\mathrm{MSE}}_{\mathrm{out}}$ and ${\mathrm{MSE}}_{\mathrm{in}}$ denote the spatial mean squared errors of the network output and the noisy input, respectively:

(23) $${\mathrm{MSE}}_{\mathrm{out}}=\frac{1}{WH}\sum_{(x,y)\in \Omega }{\left(\hat{I}\left(x,y\right)-I_{\mathrm{clean}}\left(x,y\right)\right)}^{2},$$

(24) $${\mathrm{MSE}}_{\mathrm{in}}=\frac{1}{WH}\sum_{(x,y)\in \Omega }{\left(I_{\mathrm{noisy}}\left(x,y\right)-I_{\mathrm{clean}}\left(x,y\right)\right)}^{2}.$$

Accordingly, the logarithmic denoising Gain (DG) loss is defined as

(25) $$L_{\mathrm{DG}}=\log_{10}\left(\frac{{\mathrm{MSE}}_{\mathrm{out}}}{{\mathrm{MSE}}_{\mathrm{in}}}\right).$$

This logarithmic formulation measures the residual-error ratio in the decibel-like logarithmic domain, compressing the dynamic range of the optimization target and improving the numerical stability of backpropagation in the presence of severe multiplicative fluctuations.

#### 2.2.2. Isotropic Total Variation Regularization

Although the DG loss constrains the global residual energy, it does not explicitly regulate the local spatial structure of the reconstructed image. To suppress isolated fluctuations while preserving meaningful structural transitions, an isotropic Total Variation (TV) regularization term is introduced.

Using the horizontal and vertical discrete gradient operators $\nabla_{h}$ and $\nabla_{v}$, defined as $\nabla_{h}\hat{I}\left(x,y\right)=\hat{I}\left(x+1,y\right)-\hat{I}\left(x,y\right)$ and $\nabla_{v}\hat{I}\left(x,y\right)=\hat{I}\left(x,y+1\right)-\hat{I}\left(x,y\right)$, the local isotropic gradient magnitude penalty over the entire spatial domain Ω is formulated as:

(26) $$L_{\mathrm{TV}}=\sum_{(x,y)\in \Omega }\sqrt{{\left(\nabla_{h}\hat{I}\left(x,y\right)\right)}^{2}+{\left(\nabla_{v}\hat{I}\left(x,y\right)\right)}^{2}+\epsilon }.$$

In the present work, ϵ is set to $\epsilon =10^{-8}$ to ensure numerical stability and differentiability when the local gradient magnitude approaches zero.

#### 2.2.3. β-Index Edge-Preservation Loss

To further constrain the network to preserve high-frequency edge information, a β-index-based edge-preservation term is incorporated into the loss function.

Let $\nabla^{2}I_{\mathrm{clean}}\left(x,y\right)$ and $\nabla^{2}\hat{I}\left(x,y\right)$ denote the Laplacian-filtered high-frequency components of the clean image and the reconstructed image, respectively. Their corresponding spatial mean values are defined as $\mu_{\mathrm{clean}}$ and $\mu_{\hat{I}}$. For brevity, we define the mean-removed high-frequency components as:

(27) $${\tilde{\nabla^{2}I}}_{\mathrm{clean}}\left(x,y\right)=\nabla^{2}I_{\mathrm{clean}}\left(x,y\right)-\mu_{\mathrm{clean}},$$

(28) $$\tilde{\nabla^{2}\hat{I}}\left(x,y\right)=\nabla^{2}\hat{I}\left(x,y\right)-\mu_{\hat{I}}.$$

Using these centered quantities, the cross-correlation term $C_{\beta }$ is defined by

(29) $$C_{\beta }=\sum_{(x,y)\in \Omega }{\tilde{\nabla^{2}I}}_{\mathrm{clean}}\left(x,y\right)\;\tilde{\nabla^{2}\hat{I}}\left(x,y\right).$$

Similarly, the corresponding auto-correlation energies are given by

(30) $$V_{\mathrm{clean}}=\sum_{(x,y)\in \Omega }{\left({\tilde{\nabla^{2}I}}_{\mathrm{clean}}\left(x,y\right)\right)}^{2},$$

(31) $$V_{\hat{I}}=\sum_{(x,y)\in \Omega }{\left(\tilde{\nabla^{2}\hat{I}}\left(x,y\right)\right)}^{2}.$$

Accordingly, the β index is defined as

(32) $$\beta =\frac{C_{\beta }}{\sqrt{V_{\mathrm{clean}}\;V_{\hat{I}}}}.$$

Since a larger β value indicates better consistency between the edge structures of the reconstructed image and those of the clean image, the corresponding edge-preservation loss is defined in minimization form as

(33) $$L_{\beta }=1-\beta .$$

#### 2.2.4. Final Composite Objective Function

Combining the above three components, the final composite objective function optimized during network training is formulated as

(34) $$L_{\mathrm{total}}=L_{\mathrm{DG}}+\lambda_{\mathrm{TV}}L_{\mathrm{TV}}+\lambda_{\beta }L_{\beta },$$

where $\lambda_{\mathrm{TV}}$ and $\lambda_{\beta }$ are nonnegative weighting coefficients controlling the trade-off among global residual-energy reduction, local spatial smoothness, and edge preservation. Specifically, the weighting coefficient for the Total Variation loss is empirically set to $2\times 10^{-4}$, and the coefficient for the edge-preservation term is set to 0.1. These values were determined through preliminary coarse tuning. By monitoring the visual and quantitative tradeoff between speckle suppression and structural fidelity, this specific configuration was selected to effectively prevent over-smoothing artifacts while ensuring robust edge preservation. Detailed intermediate mathematical derivations and the fully expanded formulations of the objective function are provided in Appendix A.

## 3. Experimental Results and Discussion

### 3.1. Dataset

#### 3.1.1. Simulation of Speckled Radar Images

The UC Merced Land Use dataset [51] is used to construct synthetic SAR-style speckled images. Each optical image is first regarded as a clean reflectivity map, and multiplicative coherent noise is then introduced to mimic the statistical characteristics of SAR speckle. Let $I_{\mathrm{clean}}$ denote the clean image. For each pixel, the speckle term is generated by simulating *L* independent looks of a complex scattering process. In the *k*-th look, the real and imaginary parts of the complex return are sampled from independent standard Gaussian distributions, i.e.,

(35) $$g_{k}\left(x,y\right)=u_{k}\left(x,y\right)+jv_{k}\left(x,y\right),$$

where $u_{k}\left(x,y\right)$ and $v_{k}\left(x,y\right)$ are mutually independent and follow N(0,1). The instantaneous intensity contribution of each look is then computed as

(36) $$s_{k}\left(x,y\right)=\frac{|g_{k}{\left(x,y\right)|}^{2}}{2}=\frac{u_{k}^{2}\left(x,y\right)+v_{k}^{2}\left(x,y\right)}{2}.$$

By averaging all looks, the multi-look intensity term is obtained as

(37) $$s\left(x,y\right)=\frac{1}{L}\sum_{k=1}^{L}s_{k}\left(x,y\right),$$

where *L* denotes the equivalent number of looks (ENL) [52].

Finally, the multiplicative speckle factor is defined in amplitude form as $\eta \left(x,y\right)=\sqrt{s(x,y)}$, and the simulated speckled image is generated by

(38) $$I_{\mathrm{noisy}}\left(x,y\right)=I_{\mathrm{clean}}\left(x,y\right)\eta \left(x,y\right).$$

This simulation strategy is consistent with the Nakagami/Gamma multi-look speckle model and effectively reproduces the signal-dependent fluctuations observed in coherent radar imaging. In the experiments, three ENL settings, namely L=1, L=4, and L=16, are adopted to represent strong, moderate, and weak speckle conditions, respectively. As *L* increases, the variance in the multiplicative noise decreases, resulting in progressively smoother observations. The resulting noisy–clean image pairs are used for both supervised training and controlled quantitative evaluation.

It should be noted that real radar speckle may exhibit more complex spatial correlations and system-specific artifacts that are not fully captured by the homogeneous Gamma model. Therefore, evaluation on the real-world RADIATE dataset is essential for validating the generalization capability and practical utility of the proposed model under authentic operating conditions.

#### 3.1.2. Real Radar Images

To evaluate the proposed method under authentic sensing conditions, the RADIATE dataset [53] is adopted. This dataset was collected by Heriot-Watt University using a Navtech CTS350-X millimeter-wave radar (Navtech Radar, Wantage, Oxfordshire, UK) and contains real radar scans acquired in diverse outdoor environments. The radar provides a maximum sensing range of approximately 100 m, a range resolution of 0.175 m, and an angular resolution of approximately 1.8°. The dataset covers a variety of challenging scenarios, including urban roads, rural roads, and suburban scenes, thereby offering a realistic benchmark for assessing denoising performance in complex environments.

### 3.2. Comparison Methods and Experimental Settings

To comprehensively evaluate the effectiveness of the proposed method for millimeter-wave radar image denoising, three representative denoising methods, namely PPB, SAR-BM3D, and DnCNN, are selected for comparison.

PPB (Probabilistic Patch-Based), proposed by Deledalle et al. [54], exploits a probabilistic patch similarity measure under the speckle noise model and performs denoising through iterative weighted maximum likelihood estimation. Since it is a representative model-based method specifically designed for multiplicative speckle noise, PPB is adopted as a baseline for classical statistical denoising approaches.

SAR-BM3D, proposed by Parrilli et al. [55], further incorporates nonlocal similar-patch grouping, transform-domain shrinkage, and LLMMSE-based weighting, achieving strong speckle suppression capability while preserving structural details. As one of the most representative and effective traditional SAR denoising methods, SAR-BM3D is included as a strong conventional baseline.

DnCNN, proposed by Zhang et al. [21], is a typical deep convolutional denoising model that learns the residual noise through convolutional feature extraction and residual learning. Compared with traditional model-based methods, DnCNN represents the data-driven deep learning paradigm for image denoising. Including DnCNN in the comparison helps demonstrate the effectiveness of the proposed method against a widely used deep neural network baseline.

SAR-Transformer, proposed by Perera et al. [43], exploits the self-attention mechanism of vision transformers to capture long-range spatial dependencies and global contextual information for speckle reduction. As a pioneering work applying transformer architectures to radar imagery, SAR-Transformer is included as a representative baseline for recent state-of-the-art attention-based deep learning methods.

CL-SAR, proposed by Fang et al. [42], introduces a self-supervised contrastive learning framework combined with a multi-scale despeckling network to suppress speckle without requiring clean reference images. Since it represents the latest advancements in unsupervised representation learning and contrastive optimization for real radar data, CL-SAR is adopted as a cutting-edge contemporary baseline.

Overall, these five comparison methods represent a diverse range of technical paradigms, namely probabilistic model-based denoising, nonlocal collaborative filtering, deep convolutional learning, transformer-based global attention modeling, and self-supervised contrastive learning. Therefore, they provide a highly comprehensive and representative benchmark for evaluating the proposed method against both classical and contemporary state-of-the-art approaches.

The experimental configurations used in this work are summarized in Table 1. All training and inference experiments are conducted on the same platform to ensure a fair comparison among different methods. During training, all models are optimized using the Adam optimizer with the proposed composite loss function. The batch size is set to 4, and the networks are trained for 200 epochs. Unless otherwise specified, the same experimental settings are used throughout all comparative and ablation studies.

### 3.3. Experiments on Synthetic Data

The denoising performance under synthetic noise conditions is evaluated using the UC Merced land-use dataset. The dataset comprises 1100 high-resolution images across 21 land-use categories, such as airports, urban areas, and highways, providing diverse structural and textural variations. These images are randomly partitioned into 880 training samples and 220 testing samples.

**Figure 2:**
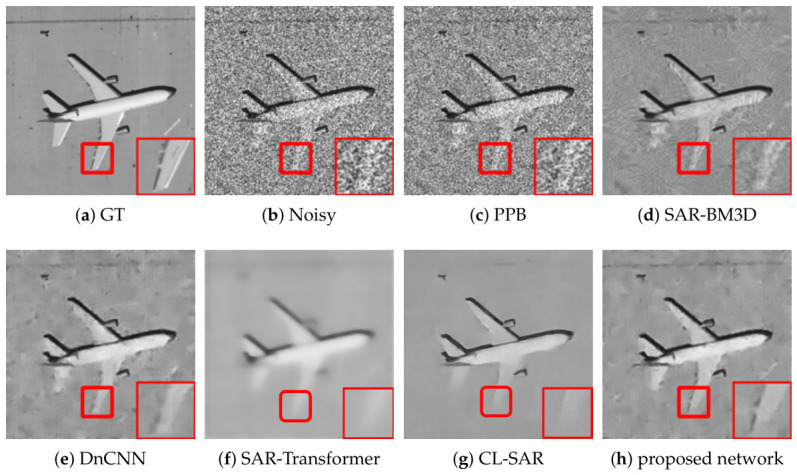
Visual comparison of speckle suppression results across different methods at ENL = 1.

**Figure 3:**
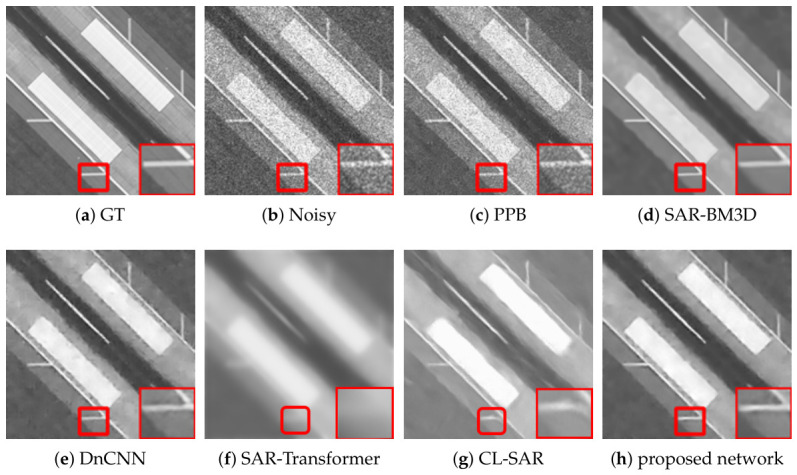
Visual comparison of speckle suppression results across different methods at ENL = 4.

**Figure 4:**
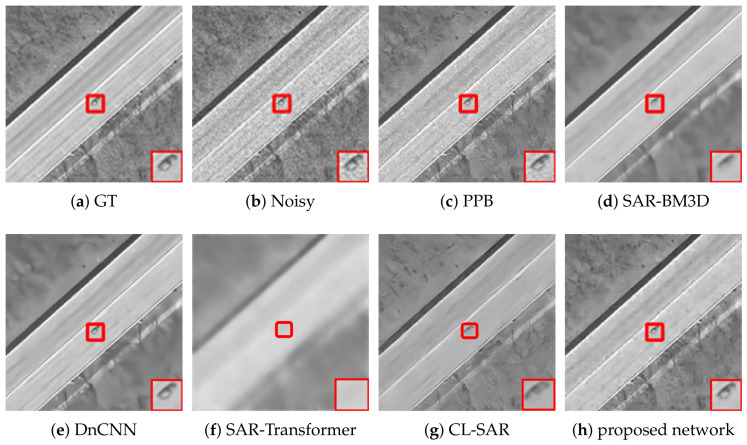
Visual comparison of speckle suppression results across different methods at ENL = 16.

Figure 2, Figure 3 and Figure 4 present a comprehensive visual comparison of the reconstructed images under three representative speckle intensities: strong, moderate, and weak degradation scenarios. By selecting complex land-use scenes containing distinct geometric structures and rich textural patterns, these visualizations explicitly demonstrate how each algorithm manages the strict trade-off between homogeneous noise suppression and high-frequency edge preservation. Specifically, the qualitative evaluation highlights the capability of the respective models to recover sharp target contours, distinct regional boundaries, and fine topological details from the severely corrupted observation data across varying noise levels.

A subjective visual analysis reveals the limitations of the compared denoising methods. Specifically, PPB and DnCNN fail to provide sufficient speckle suppression, leaving noticeable residual noise in homogeneous regions. In contrast, SAR-BM3D achieves stronger noise reduction, but in the ENL = 16 case, it tends to introduce over-smoothing, which weakens edge sharpness and suppresses fine structural details. Furthermore, although SAR-Transformer effectively suppresses speckle, it severely sacrifices overall image clarity and renders the structural boundaries blurry. Similarly, while CL-SAR achieves aggressive noise reduction, it inevitably destroys fine texture details and topological features. By comparison, the proposed deep learning network achieves a more favorable balance between noise suppression and detail preservation, effectively reducing speckle while maintaining structural integrity and edge continuity.

It should be noted that the red boxes in Figure 2, Figure 3 and Figure 4 are not only used to highlight representative local regions for visual comparison but also serve as the regions of interest for SSIM evaluation. In other words, the local SSIM values are computed on the red-boxed patches to quantitatively assess structural similarity preservation in critical detail regions. This design makes the SSIM measurement more focused on informative structures, such as edges, boundaries, and small targets, rather than being dominated by large homogeneous background areas.

As summarized in Table 2, the proposed deep learning network demonstrates a clear and consistent performance improvement as the ENL increases from 1 to 16. Specifically, the average PSNR increases from 24.49 dB to 30.43 dB, corresponding to an absolute gain of 5.94 dB, while the average SSIM increases from 0.675 to 0.868, with an absolute improvement of 0.193. These results indicate that, as the speckle level becomes weaker, the proposed model can recover image structures more faithfully and produce higher-quality restorations. Overall, the quantitative results further confirm the robustness and effectiveness of the proposed deep learning network under different noise conditions.

Table 3 summarizes the quantitative results on the UC Merced dataset using PSNR and SSIM under different speckle levels. A quantitative analysis reveals that the structural-prior deep learning network achieves a highly competitive and robust balance between noise reduction and structural preservation across various degradation intensities.

Under severe speckle noise with an equivalent number of looks equal to one, the proposed network achieves 27.35 dB in PSNR and 0.681 in SSIM. Although the recent CL-SAR baseline yields a slightly higher SSIM of 0.693, its exceptionally low PSNR of 11.53 dB indicates severe global signal degradation and intensity distortion. Consequently, the proposed architecture successfully maintains structural fidelity without sacrificing overall image quality.

Under moderate noise conditions with an equivalent number of looks equal to four, the proposed network still provides the best reconstruction quality, with the highest PSNR of 27.76 dB and the highest SSIM of 0.735. Even in the relatively mild noise case where the equivalent number of looks equals sixteen, the proposed network remains superior, reaching 29.10 dB and 0.794 in PSNR and SSIM, respectively.

Compared with classical filters and recent deep learning models including SAR-Transformer and CL-SAR, the stronger comprehensive performance of our method across all settings indicates that the proposed architecture is more effective at preserving structural information and perceptual consistency while removing speckle. These results confirm the robustness and generalization ability of the proposed structural-prior deep learning network under different noise intensities.

### 3.4. Experiments on Real Radar Data

While synthetic evaluations provide a controlled baseline, they rely on idealized speckle distributions that inherently fail to capture the complex, heterogeneous clutter of practical radar imaging. To bridge this gap and thoroughly demonstrate real-world generalization, we extend our validation to the RADIATE dataset. It is important to emphasize that our proposed network was strictly trained on the synthetic UC Merced dataset and was never fine-tuned on the RADIATE dataset. Consequently, all performance evaluations on the real-world RADIATE data represent direct cross-domain inference. This transfer capability explicitly validates the strong generalization robustness of the proposed structural-prior-guided architecture in handling unseen authentic radar clutter. By evaluating representative scans from rural, suburban, and densely urban scenarios, we test the network’s performance against highly diverse structural complexities and clutter profiles. To ensure the statistical validity and scientific rigor of evaluating the equivalent number of looks on real-world millimeter-wave radar data, we strictly confined the calculation to highly homogeneous local regions. Across diverse scenarios, including both urban and suburban road scenes, flat asphalt surfaces devoid of vehicles, lane markings, and strong corner reflectors were manually selected. This rigorous selection protocol effectively isolates the residual speckle variance from the underlying structural geometry. Consequently, the measured ENL accurately reflects genuine noise suppression capabilities, rather than an artificial inflation caused by over-smoothing target details.

**Figure 5:**
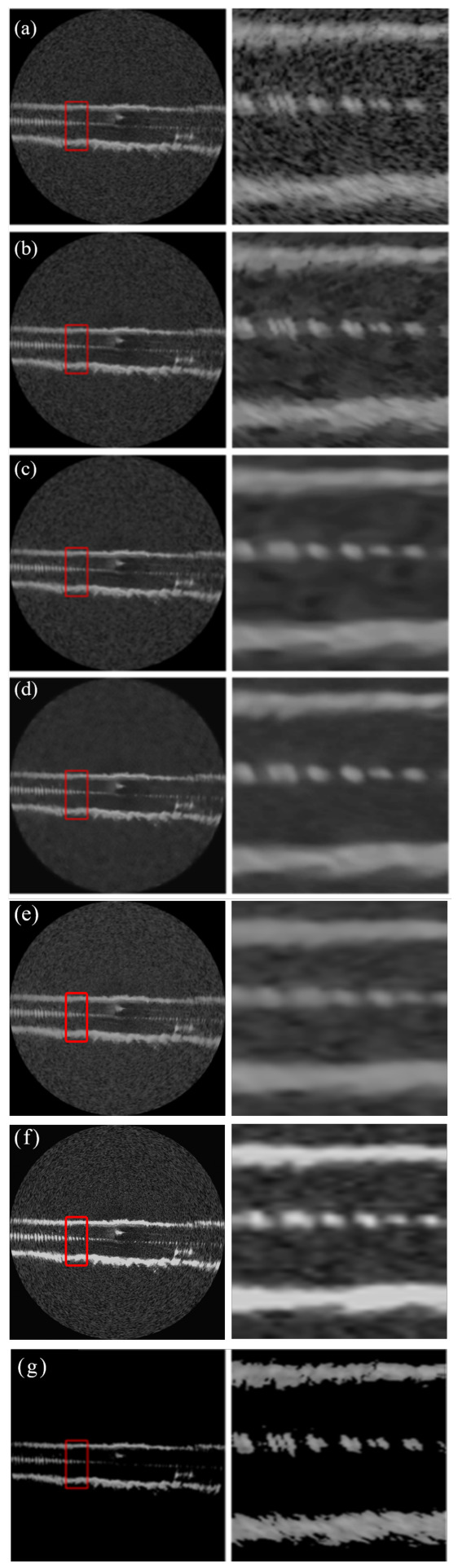
Denoising results on rural road scenes. The left column shows the full radar images, while the right column shows enlarged views of the regions highlighted by the red boxes in the corresponding left-column images. (**a**) Noisy image, (**b**) PPB, (**c**) SAR-BM3D, (**d**) DnCNN, (**e**) SAR-Transformer, (**f**) CL-SAR, (**g**) proposed network.

**Figure 6:**
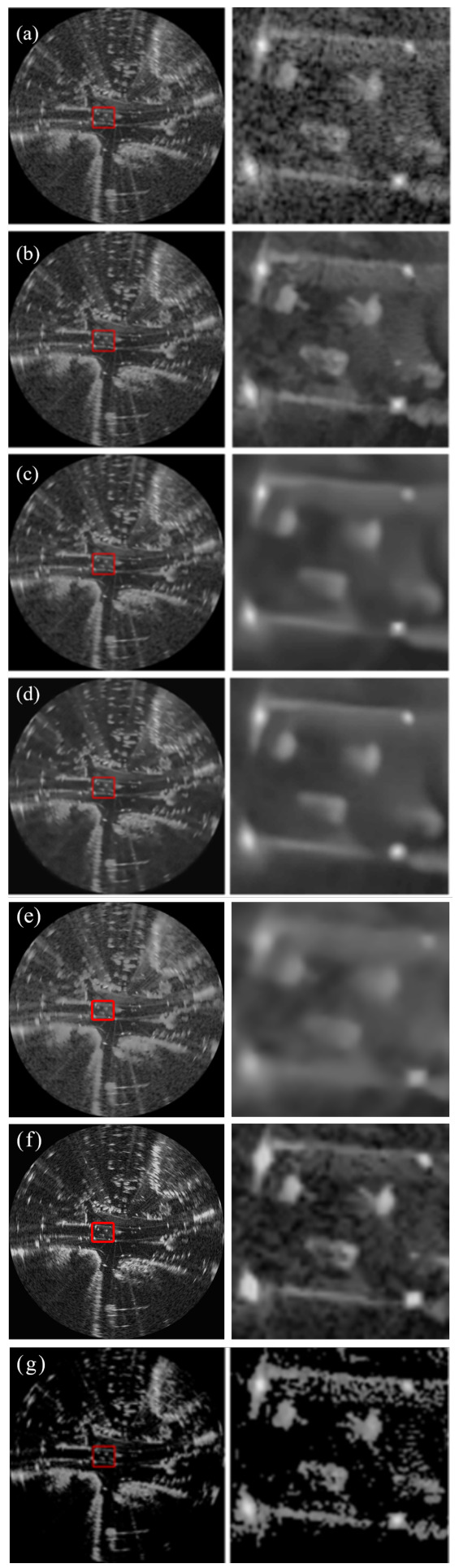
Denoising results on urban road scenes. The left column shows the full radar images, while the right column shows enlarged views of the regions highlighted by the red boxes in the corresponding left-column images. (**a**) Noisy image, (**b**) PPB, (**c**) SAR-BM3D, (**d**) DnCNN, (**e**) SAR-Transformer, (**f**) CL-SAR, (**g**) proposed network.

**Figure 7:**
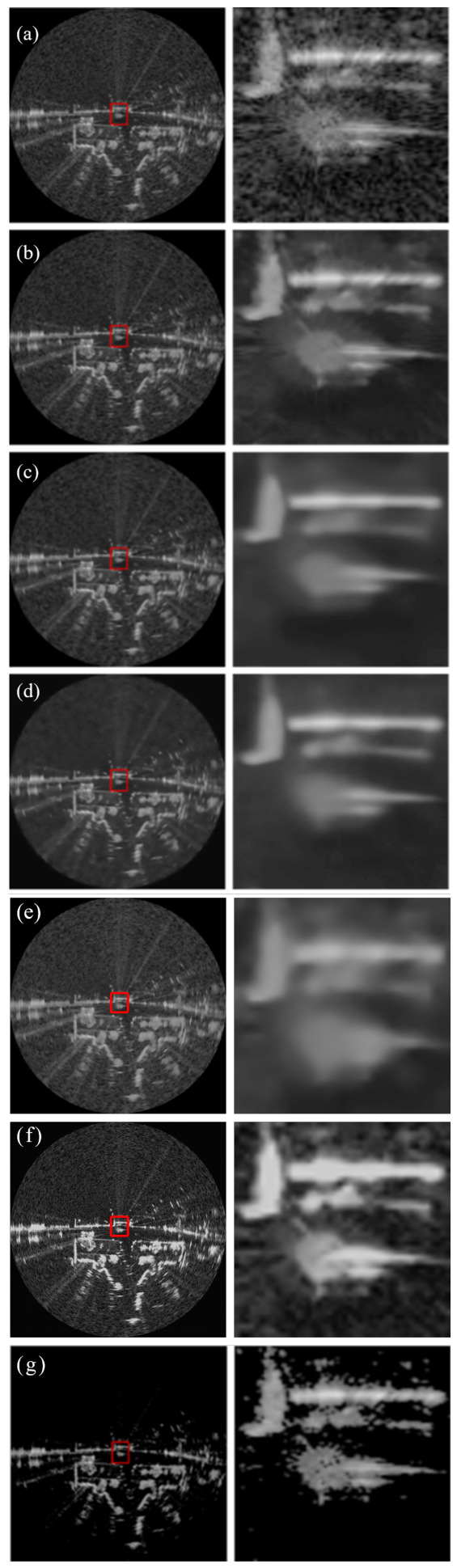
Denoising results on suburban road scenes. The left column shows the full radar images, while the right column shows enlarged views of the regions highlighted by the red boxes in the corresponding left-column images. (**a**) Noisy image, (**b**) PPB, (**c**) SAR-BM3D, (**d**) DnCNN, (**e**) SAR-Transformer, (**f**) CL-SAR, (**g**) proposed network.

Figure 5, Figure 6 and Figure 7 present the denoising results on real radar images collected in three representative scenarios, namely suburban, urban, and rural roads. Across all three scenes, the proposed deep learning network achieves a more favorable balance between speckle suppression and structural preservation than the compared methods.

In the rural scene shown in Figure 5, the radar echoes are mainly characterized by elongated road boundaries and regularly distributed bright scattering points. PPB reduces part of the background fluctuation, but residual speckle remains evident, and the local structures are still blurred. SAR-BM3D and DnCNN further suppress the noise, yet both methods introduce excessive smoothing in the enlarged region, causing the weak linear structures and discrete scattering responses to become less distinguishable. Additionally, while SAR-Transformer achieves a smooth background, it severely blurs the elongated road boundaries. Meanwhile, CL-SAR suppresses noise aggressively but introduces noticeable topological distortions to the parallel linear features. In contrast, the proposed deep learning network preserves the parallel boundary structures and the aligned bright scattering points more clearly while significantly reducing the background speckle. This indicates that the proposed method can better maintain fine structural continuity in relatively regular road scenes.

As illustrated in the urban scene of Figure 6, the background becomes much more complicated because of dense clutter, strong multi-path reflections, and numerous heterogeneous scatterers. Under this condition, the enlarged region contains richer local details and more irregular high-intensity responses. PPB leaves noticeable residual noise, whereas SAR-BM3D and DnCNN tend to oversmooth the scene and blur the boundaries of salient targets. Furthermore, SAR-Transformer sacrifices overall image clarity and renders complex urban targets into blurry regions, and CL-SAR severely distorts the fine textures of heterogeneous scatterers. By comparison, the proposed deep learning network still retains lane-like boundaries, corner-like bright responses, and local scattering structures with higher contrast, demonstrating stronger robustness in highly cluttered and structurally complex environments.

In the suburban scene shown in Figure 7, the scene contains sparse but prominent scattering structures, together with extended background fluctuations. Although the overall clutter density is lower than that in the urban case, the target contours and linear features are relatively weak and can be oversmoothed easily during denoising. PPB provides limited noise suppression, while SAR-BM3D and DnCNN remove substantial speckle at the cost of attenuating important structural information in the highlighted region. Similarly, SAR-Transformer over-smooths the prominent scattering structures, and CL-SAR corrupts the topological integrity of the weak linear features. In contrast, the proposed deep learning network preserves the main scattering contours and elongated responses more effectively while maintaining a cleaner background. This suggests that the proposed method is also capable of handling scenes with sparse structures and low-texture backgrounds.

Overall, the visual comparisons across suburban, urban, and rural environments verify that the proposed deep learning network exhibits stronger adaptability to different real radar scenes. Compared with the competing methods, it more consistently suppresses speckle while preserving meaningful structural details, especially in the enlarged local regions marked by the red boxes.

Table 4 compares the β-index and ENL values across three real radar scenes. The β-index quantitatively measures the preservation of edge contrast after denoising, reflecting the ability of a method to suppress speckle while maintaining sharp structural transitions. The ENL characterizes the smoothness in homogeneous regions, where a higher value typically indicates stronger speckle suppression. This metric is especially relevant for radar-based perception tasks, where edge integrity directly affects downstream object detection and scene understanding.

A quantitative analysis reveals a complex trade-off between noise reduction and structural fidelity among the evaluated methods. Although SAR-Transformer achieves the highest ENL values, a visual inspection indicates that this is accomplished at the cost of severe blurring artifacts, which obscure critical road details. Similarly, while CL-SAR exhibits high β-index scores, it tends to forcefully smooth edge regions, resulting in noticeable image distortion and the loss of essential texture details. By contrast, the proposed Network achieves the most favorable balance across all scenarios. It effectively suppresses background clutter while maintaining the highest degree of structural realism and boundary clarity, thereby providing a more reliable foundation for downstream perception tasks.

The robust performance of the proposed deep learning network in complex scenarios is significantly driven by the Otsu-based masking module. Acting as an early hard-attention filter, it explicitly isolates highly reflective targets from dense background clutter. This crucial separation prevents non-stationary noise from corrupting training gradients, enabling the continuous attention flow to focus exclusively on preserving high-frequency structural details. Consequently, the proposed architecture effectively circumvents the visual distortion and over-smoothing degradation typical of conventional and recent SOTA methods.

### 3.5. Ablation Study

#### 3.5.1. Evaluation on Synthetic Data

The first tier of the ablation study is conducted on the synthetic UC Merced dataset at a noise level of L = 4. Because synthetic data possesses absolute noise-free reference images, it allows for strict pixel-level and structural fidelity evaluation using full-reference metrics, namely PSNR and SSIM. Table 5 reports the performance under eight different module configurations.

The baseline model lacking all attention modules yields the lowest PSNR and SSIM. The integration of individual modules progressively enhances restoration fidelity. Notably, the CB expands the receptive field, the CBAM highlights salient regions, and the RCAB bolsters inter-channel feature recalibration. Furthermore, explicitly ablating the Otsu structural prior layer while retaining all deep attention modules results in a noticeable performance drop, with PSNR decreasing from 31.38 dB to 30.37 dB. This decline confirms that early structural guidance is critical for preventing non-stationary noise from corrupting the deep feature representations. The complete Structural-Prior Deep Learning Network architecture achieves the highest PSNR of 31.38 dB, demonstrating superior theoretical noise suppression under controlled Gamma distribution conditions.

#### 3.5.2. Evaluation on Real Radar Data

While synthetic evaluation is fundamental, real-world millimeter-wave radar data exhibits complex and spatially variant clutter while inherently lacking clean reference images. Therefore, the second tier evaluates the modules on the real-world RADIATE dataset for urban road scenarios using established no-reference metrics. Specifically, the ENL is utilized to measure homogeneous noise suppression, and the β-index is employed to quantify edge preservation capability. To ensure the scientific rigor of this ENL assessment amidst heterogeneous urban clutter, we strictly confined the calculation to highly homogeneous local regions. Flat asphalt surfaces devoid of vehicles, lane markings, and strong corner reflectors were manually selected to isolate the residual speckle variance from the underlying scene geometry. This rigorous protocol guarantees that the reported ENL reflects genuine noise suppression, rather than an artificial inflation caused by over-smoothing target details.

Consistent with the synthetic results, Table 6 reveals that the synergy of all four modules is essential for practical deployment. Crucially, when the Otsu structural prior layer is removed, the edge preservation metric drops to 0.427 and the background smoothness index decreases to 28.3. This quantitative decline validates our hypothesis that a preliminary separation of salient targets from background clutter is fundamentally necessary to prevent the subsequent continuous attention flow from allocating excessive modeling capacity to noise. The omission of the spatial-channel joint attention module results in the sharpest decline in both edge preservation and overall smoothing among the deep feature blocks. Specifically, the β-index drops from 0.445 to 0.360, and the ENL drops from 30.2 to 22.4. This dual-tier evaluation solidifies the conclusion that the integration of structural-prior guidance and the proposed continuous attention flow is robust and indispensable for both theoretical fidelity and practical interpretability in complex radar scenes.

### 3.6. Discussion on Domain Shift and Generalization Analysis

It is essential to discuss the training-to-testing paradigm of the Structural-Prior Deep Learning Network, which inherently involves a significant domain shift. Since acquiring absolutely clean reference images for real-world millimeter-wave radar is fundamentally impossible, the network is trained exclusively on high-resolution optical images from the UC Merced dataset corrupted by simulated multiplicative speckle. Consequently, applying this pre-trained model to the authentic RADIATE dataset constitutes a zero-shot cross-domain inference task.

The successful generalization of the proposed architecture is primarily attributed to the explicit structural prior and the geometry-aware composite loss function. By forcing the network to focus on invariant topological features and structural boundaries, rather than merely overfitting to the synthetic noise distribution, these components effectively bridge the gap between optical simulations and real-world radar sensing. This paradigm demonstrates that structural-prior guidance provides a robust and practical solution for radar image enhancement when authentic training labels are unavailable.

### 3.7. Computational Complexity Analysis

To evaluate the practical applicability of the proposed network for real-time road sensing, we provide a detailed comparison of computational complexity. The evaluation includes the number of trainable parameters, multiply-accumulate operations in GMac, and the average inference time per image. All measurements were conducted on a hardware platform equipped with an NVIDIA GeForce RTX 4060 GPU.

The comparative analysis in Table 7 highlights the efficiency of the Structural-Prior Deep Learning Network across different technical paradigms. Traditional model-based methods such as PPB and SAR-BM3D involve no trainable parameters, but their inference latencies are significantly higher due to intensive iterative optimization processes. Among deep learning models, while SAR-Transformer exhibits a low multiply-accumulate metric, it suffers from a massive parameter burden exceeding 24 million. This huge memory footprint significantly hinders its deployment on memory-constrained edge devices in autonomous vehicles. Conversely, CL-SAR incurs excessive computational latency requiring 82.80 milliseconds per frame. By contrast, the Structural-Prior Deep Learning Network maintains a lightweight parameter count of 3.359 million and achieves a rapid inference time of 12.35 milliseconds. This optimal balance of memory footprint and processing speed effectively circumvents the deployment bottlenecks typical of recent state-of-the-art models, satisfying the real-time perception requirements of autonomous driving systems.

## 4. Conclusions

This paper presented a structural-prior-guided deep learning framework for millimeter-wave radar image enhancement in autonomous driving road perception. To address severe background interference and signal-dependent multiplicative speckle, the proposed method incorporates an image structural-prior layer into a unified encoder–decoder architecture with continuous attention-based feature refinement. By introducing prior structural guidance before deep feature transformation and combining progressive feature enhancement with global information preservation, the proposed network effectively suppresses speckle contamination while maintaining salient target structures, boundary information, and scene geometry. In addition, a composite loss function integrating logarithmic denoising gain, total variation regularization, and a β-index edge-preservation term was designed to jointly improve noise suppression, spatial smoothness, and structural fidelity.

Experimental results on both synthetic and real millimeter-wave radar datasets demonstrated that the proposed method consistently outperformed the compared baseline methods, including recent transformer-based models, in terms of PSNR, SSIM, and β-index. Qualitative comparisons further showed that the proposed framework achieved a more favorable tradeoff between interference suppression and structural preservation across different road environments. Ablation studies also verified the effectiveness of the structural-prior guidance and continuous attention design in improving restoration performance. Furthermore, computational complexity analysis confirmed that the proposed network maintains a lightweight profile with merely 3.359 million parameters and a rapid inference latency of 12.35 milliseconds, effectively overcoming the massive memory burden typical of attention-heavy architectures. Overall, the proposed framework provides an effective and robust solution for millimeter-wave radar image enhancement and offers practical value for downstream road-scene perception in autonomous driving scenarios.

## Figures and Tables

**Table 1:** Experimental configuration.

Parameter	Setting
Operating System	Windows 11
CPU	12th Gen Intel Core i5-12490F @ 3.00 GHz
Memory	32 GB
GPU	NVIDIA GeForce RTX 4060
Python Version	Python 3.7
Deep Learning Framework	PyTorch 1.13

**Table 2:** Average quantitative performance of the learning network under different ENL levels.

ENL	PSNR (dB)	SSIM
1	24.49	0.675
4	27.35	0.784
16	30.43	0.868

**Table 3:** Denoising results on UC Merced dataset.

Method	ENL = 1		ENL = 4		ENL = 16	
	PSNR	SSIM	PSNR	SSIM	PSNR	SSIM
PPB [54]	13.33	0.094	19.90	0.374	25.84	0.624
SAR-BM3D [55]	23.15	0.374	27.64	0.697	28.14	0.663
DnCNN [21]	25.68	0.592	26.41	0.649	28.93	0.713
SAR-Transformer [43]	16.7	0.153	20.08	0.43	20.43	0.623
CL-SAR [42]	11.53	0.693	10.20	0.507	13.40	0.667
proposed network	27.35	0.681	27.76	0.735	29.10	0.794

**Table 4:** Quantitative evaluation results on real radar images.

Method	Rural Road		Urban Road		Suburban Road	
	β-Index	ENL	β-Index	ENL	β-Index	ENL
PPB [54]	0.517	2.608	0.432	3.288	0.454	3.065
SAR-BM3D [55]	0.294	2.719	0.245	3.765	0.227	3.444
DnCNN [21]	0.269	0.790	0.221	0.736	0.209	0.612
SAR-Transformer [43]	0.163	3.503	0.161	5.638	0.153	5.018
CL-SAR [42]	0.757	3.313	0.712	3.020	0.785	2.865
proposed network	0.521	0.701	0.445	0.376	0.479	0.297

**Table 5:** Ablation study of different module combinations on the synthetic UC Merced dataset.

Module Configuration				Metrics	
OTSU	RCAB	CB	CBAM	PSNR	SSIM
+	−	−	−	28.12	0.832
+	+	−	−	29.54	0.851
+	−	+	−	29.03	0.843
+	−	−	+	29.78	0.857
+	+	+	−	30.32	0.865
+	+	−	+	30.92	0.881
+	−	+	+	30.65	0.873
−	+	+	+	30.37	0.865
+	+	+	+	31.38	0.887

**Table 6:** Ablation study of different module combinations on the real-world RADIATE dataset for urban scenarios.

Module Configuration				Metrics	
OTSU	RCAB	CB	CBAM	β-Index	ENL
+	−	−	−	0.250	15.2
+	+	−	−	0.310	18.5
+	−	+	−	0.295	17.8
+	−	−	+	0.335	20.1
+	+	+	−	0.360	22.4
+	+	−	+	0.410	26.7
+	−	+	+	0.395	25.3
−	+	+	+	0.427	28.3
+	+	+	+	0.445	30.2

**Table 7:** Comparison of computational complexity and inference efficiency.

Method	Parameters in M	FLOPs in GMac	Inference Time in ms
PPB [54]	—	—	1250.4
SAR-BM3D [55]	—	—	3420.8
DnCNN [21]	0.556	36.58	7.41
SAR-Transformer [43]	24.099	5.75	13.94
CL-SAR [42]	10.038	95.45	82.80
propose network	3.359	51.29	12.35

## Data Availability

Due to the large volume of the dataset, the data presented in this study are available from the corresponding author upon reasonable request.

## Abbreviations

BEV	Bird’s-Eye View
CAB	Channel Attention Block
CAM	Channel Attention Module
CB	Context-Aware Block
CBAM	Convolutional Block Attention Module
CNN	Convolutional Neural Network
CPU	Central Processing Unit
DnCNN	Denoising Convolutional Neural Network
ENL	Equivalent Number of Looks
FFDNet	Fast and Flexible Denoising Network
GANs	Generative Adversarial Networks
GMac	Giga Multiply-Accumulate Operations
GPU	Graphics Processing Unit
LiDAR	Light Detection and Ranging
LLMMSE	Linear Local Minimum Mean Square Error
MAP	Maximum A Posteriori
MLP	Multi-Layer Perceptron
mmWave	Millimetre Wave
MSE	Mean Squared Error
MWR	Millimetre-Wave Radar
OTSU	Otsu Thresholding
PDF	Probability Density Function
PPB	Probabilistic Patch-Based
PSNR	Peak Signal-to-Noise Ratio
RADIATE	RADIATE Dataset
RCAB	Residual Channel Attention Block
ReLU	Rectified Linear Unit
SAM	Spatial Attention Module
SAR	Synthetic Aperture Radar
SAR-BM3D	Synthetic Aperture Radar Block-Matching and 3D Filtering
SSIM	Structural Similarity Index Measure
TV	Total Variation

## Appendix A. Detailed Mathematical Derivations of the Composite Loss Function

To maintain the compactness of the main text, this appendix provides the detailed intermediate derivations and the fully expanded mathematical formulations for the composite objective function presented in Section 2.2.

### Appendix A.1. Derivation of the Logarithmic Denoising Gain Loss

To formulate the residual-energy reduction, we first define the output residual and the input residual at pixel (x,y) as:

(A1) $$e_{\mathrm{out}}\left(x,y\right)=\hat{I}\left(x,y\right)-I_{\mathrm{clean}}\left(x,y\right),\;e_{\mathrm{in}}\left(x,y\right)=I_{\mathrm{noisy}}\left(x,y\right)-I_{\mathrm{clean}}\left(x,y\right).$$

Based on these residuals, the corresponding spatial residual energies over the whole image are explicitly defined by summing over the entire spatial domain Ω:

(A2) $$E_{\mathrm{out}}=\sum_{(x,y)\in \Omega }{\left(\hat{I}\left(x,y\right)-I_{\mathrm{clean}}\left(x,y\right)\right)}^{2},$$

(A3) $$E_{\mathrm{in}}=\sum_{(x,y)\in \Omega }{\left(I_{\mathrm{noisy}}\left(x,y\right)-I_{\mathrm{clean}}\left(x,y\right)\right)}^{2}.$$

By substituting the energy equations into the logarithmic gain definition, the Logarithmic Denoising Gain loss can be expressed in its explicit pixel-wise expanded form as:

(A4) $$L_{\mathrm{DG}}=\log_{10}\left(\frac{\sum_{(x,y)\in \Omega }{\left(\hat{I}\left(x,y\right)-I_{\mathrm{clean}}\left(x,y\right)\right)}^{2}}{\sum_{(x,y)\in \Omega }{\left(I_{\mathrm{noisy}}\left(x,y\right)-I_{\mathrm{clean}}\left(x,y\right)\right)}^{2}}\right).$$

This formulation is mathematically identical to the ratio of Mean Squared Errors defined in Equation (25), as the normalization factor WH cancels out.

### Appendix A.2. Expansion of the Isotropic Total Variation Regularization

The horizontal and vertical first-order forward differences of the reconstructed image at pixel (x,y) are defined as $d_{h}\left(x,y\right)=\hat{I}\left(x+1,y\right)-\hat{I}\left(x,y\right)$ and $d_{v}\left(x,y\right)=\hat{I}\left(x,y+1\right)-\hat{I}\left(x,y\right)$. The corresponding local gradient vector is given by $d\left(x,y\right)={\left[d_{h}\left(x,y\right),d_{v}\left(x,y\right)\right]}^{T}$.

Based on this, the local isotropic gradient magnitude penalty g(x,y) is explicitly formulated as:

(A5) $$g\left(x,y\right)=\sqrt{d_{h}^{2}\left(x,y\right)+d_{v}^{2}\left(x,y\right)+\epsilon }=\sqrt{{∥d\left(x,y\right)∥}_{2}^{2}+\epsilon }.$$

Summing this local penalty over the entire spatial domain yields the explicit form of the TV loss:

(A6) $$L_{\mathrm{TV}}=\sum_{(x,y)\in \Omega }\sqrt{{\left(\hat{I}\left(x+1,y\right)-\hat{I}\left(x,y\right)\right)}^{2}+{\left(\hat{I}\left(x,y+1\right)-\hat{I}\left(x,y\right)\right)}^{2}+\epsilon }.$$

### Appendix A.3. Explicit Formulation of the β-Index

The spatial mean values of the Laplacian-filtered high-frequency components are explicitly calculated as:

(A7) $$\mu_{\mathrm{clean}}=\frac{1}{WH}\sum_{(x,y)\in \Omega }\nabla^{2}I_{\mathrm{clean}}\left(x,y\right),\;\mu_{\hat{I}}=\frac{1}{WH}\sum_{(x,y)\in \Omega }\nabla^{2}\hat{I}\left(x,y\right).$$

By substituting the cross-correlation and auto-correlation energy formulations into the β index definition, the explicit full-image form becomes:

(A8) $$\beta =\frac{\sum_{(x,y)\in \Omega }\left(\nabla^{2}I_{\mathrm{clean}}\left(x,y\right)-\mu_{\mathrm{clean}}\right)\left(\nabla^{2}\hat{I}\left(x,y\right)-\mu_{\hat{I}}\right)}{\sqrt{\left[\sum_{(x,y)\in \Omega }{\left(\nabla^{2}I_{\mathrm{clean}}\left(x,y\right)-\mu_{\mathrm{clean}}\right)}^{2}\right]\left[\sum_{(x,y)\in \Omega }{\left(\nabla^{2}\hat{I}\left(x,y\right)-\mu_{\hat{I}}\right)}^{2}\right]}}.$$

### Appendix A.4. Fully Expanded Composite Objective Function

By substituting the explicit formulations of the DG loss, the TV loss, and the β-index loss into the final objective function, the complete and fully expanded form of the composite loss optimized during network training is given by:

(A9) $$\begin{array}{cc} L_{\mathrm{total}} & =\log_{10}\left(\frac{\sum_{(x,y)\in \Omega }{\left(\hat{I}\left(x,y\right)-I_{\mathrm{clean}}\left(x,y\right)\right)}^{2}}{\sum_{(x,y)\in \Omega }{\left(I_{\mathrm{noisy}}\left(x,y\right)-I_{\mathrm{clean}}\left(x,y\right)\right)}^{2}}\right)\\ \; & \;+\lambda_{\mathrm{TV}}\sum_{(x,y)\in \Omega }\sqrt{{\left(\hat{I}\left(x+1,y\right)-\hat{I}\left(x,y\right)\right)}^{2}+{\left(\hat{I}\left(x,y+1\right)-\hat{I}\left(x,y\right)\right)}^{2}+\epsilon }\\ \; & \;+\lambda_{\beta }\left[1-\frac{\sum_{(x,y)\in \Omega }\left(\nabla^{2}I_{\mathrm{clean}}\left(x,y\right)-\mu_{\mathrm{clean}}\right)\left(\nabla^{2}\hat{I}\left(x,y\right)-\mu_{\hat{I}}\right)}{\sqrt{\left[\sum_{(x,y)\in \Omega }{\left(\nabla^{2}I_{\mathrm{clean}}\left(x,y\right)-\mu_{\mathrm{clean}}\right)}^{2}\right]\left[\sum_{(x,y)\in \Omega }{\left(\nabla^{2}\hat{I}\left(x,y\right)-\mu_{\hat{I}}\right)}^{2}\right]}}\right]. \end{array}$$
